# Transcription and Translation Inhibitors in Cancer Treatment

**DOI:** 10.3389/fchem.2020.00276

**Published:** 2020-04-21

**Authors:** Nihay Laham-Karam, Gaspar P. Pinto, Antti Poso, Piia Kokkonen

**Affiliations:** ^1^A. I. Virtanen Institute for Molecular Sciences, University of Eastern Finland, Kuopio, Finland; ^2^International Clinical Research Center, St. Anne University Hospital, Brno, Czechia; ^3^Loschmidt Laboratories, Department of Experimental Biology and RECETOX, Faculty of Science, Masaryk University, Brno, Czechia; ^4^School of Pharmacy, Faculty of Health Sciences, University of Eastern Finland, Kuopio, Finland; ^5^University Hospital Tübingen, Department of Internal Medicine VIII, University of Tübingen, Tübingen, Germany

**Keywords:** cancer, drug, inhibitor, translation, transcription

## Abstract

Transcription and translation are fundamental cellular processes that govern the protein production of cells. These processes are generally up regulated in cancer cells, to maintain the enhanced metabolism and proliferative state of these cells. As such cancerous cells can be susceptible to transcription and translation inhibitors. There are numerous druggable proteins involved in transcription and translation which make lucrative targets for cancer drug development. In addition to proteins, recent years have shown that the “undruggable” transcription factors and RNA molecules can also be targeted to hamper the transcription or translation in cancer. In this review, we summarize the properties and function of the transcription and translation inhibitors that have been tested and developed, focusing on the advances of the last 5 years. To complement this, we also discuss some of the recent advances in targeting oncogenes tightly controlling transcription including transcription factors and KRAS. In addition to natural and synthetic compounds, we review DNA and RNA based approaches to develop cancer drugs. Finally, we conclude with the outlook to the future of the development of transcription and translation inhibitors.

## Introduction

Cancer refers to a large group of diseases of uncontrolled cell growth and division where a general cure or containment is nowhere to be seen. As a testament to this, the research in cancer is ongoing vigorously and journals specifically tailored for cancer research are now more than 50, ranging from the general to the specific types of cancer. According to the World Health Organization, the various types of cancer accounted for nearly 10 million deaths in 2018, which made it the second most common cause of death. The common factor in cancers is the malignant transformation of cells due to acquired genetic mutations. These are often many and can include both driver and passenger mutations, that confer a growth advantage (Pon and Marra, 2015). These mutations can lead to activation (gain of function) or deactivation (loss of function,) in some of the biological processes that lead to cancer. When transcriptional or translational processes are disrupted a tumor might be formed.

Transcription can be divided into four stages. It starts with the pre-initiation complex (PIC) and is followed by initiation, elongation and termination of the process (Roeder, 1996). The initiation is achieved when the RNA polymerase (Pol) II and transcription factors are complexed with a mediator that helps to stabilize them (Kornberg, 2005; Plaschka et al., 2015). Following the formation of this complex, the elongation is initiated with the help of proteins known as activators and repressors. The elongation, the creation of an RNA copy of the DNA sequence, terminates when polyadenylation occurs, a process that is yet to be totally understood (Lykke-Andersen and Jensen, 2007; Watson et al., 2013; Proudfoot, 2016). The transcription is followed by the post-transcriptional process where RNA-binding proteins play an important role. Their role in cancer and the way that they are dysregulated in several types of cancer have been reviewed elsewhere (Pereira et al., 2017a).

Translation refers to the process of protein synthesis according to the mRNA template. It is a well-controlled process that includes not only mRNA but also tRNA, ribosomes and transcription and elongation factors (Sonenberg and Hinnebusch, 2009). Like transcription, the translation process is divided into four steps, starting with the initiation, followed by elongation and termination of the process, and to finalize translation the ribosome is recycled. A controlled translation process is required for protein synthesis and normal cell cycling (Vogel and Marcotte, 2012; Kristensen et al., 2013). When the translation process is dysregulated and there are gain of function disruptions, protein synthesis increases which leads to tumor growth.

There are several different approaches for the treatment of cancers (Arruebo et al., 2011). Nowadays, almost always two or more cancer therapies are used in combination to decrease the possibility of developing resistance (Flaherty, 2006). Surgery to remove as much as possible of the cancerous growth is usually the first way of treatment for solid tumors. Radiation and chemotherapy can be used to destroy cancer cells which cannot be removed by surgery. The newest addition to our cancer treatment methods are immunotherapy and oncological virus therapies that make use of the patient's own immune system to attack the cancer cells (Schirrmacher, 2018). The hematological malignancies such as leukemias and lymphomas can benefit from bone marrow therapy where own or donor hematopoietic stem cells are transplanted into the patient to replace the diseased cells (Simpson and Dazzi, 2019).

Chemotherapy is a general term used for all chemicals to treat cancer. It includes for example hormone therapy, which is used to slow down hormone-reactive cancer growth, and cisplatin which prevents the replication of DNA. Also, the focus of this review, targeted therapies are included under chemotherapy. Targeted therapy differs from general chemotherapy by taking a more specialized approach which can be compared to a sniper rifle (targeted therapy) vs. a shotgun (chemotherapy). In other words, the probability of killing healthy cells is lower with targeted therapies than with general chemotherapeutics. While chemotherapy is directed to the inhibition of cell mitosis or inducing autophagy, targeted small molecules inhibitors act on the transcription and translation processes.

Transcription and translation offer great possibilities and dozens of potential targets for developing drugs against cancer. Despite these promises, the efforts to produce such drugs have been hindered by our limited understanding of the underlying biology, cancers developing resistance and a myriad of alternative pathways that ensure the function of these crucial pathways (Villicaña et al., 2014; Bhat et al., 2015). In this review, we give a general overview of the approaches used in the inhibition of the transcription and the translation with the goal to treat cancer. Since there are a few reviews of similar topics from earlier years, we will be focusing on the advantages of the last 5 years (Stellrecht and Chen, 2011; Villicaña et al., 2014; Bhat et al., 2015).

## Targeting Transcription

**Figure 1:**
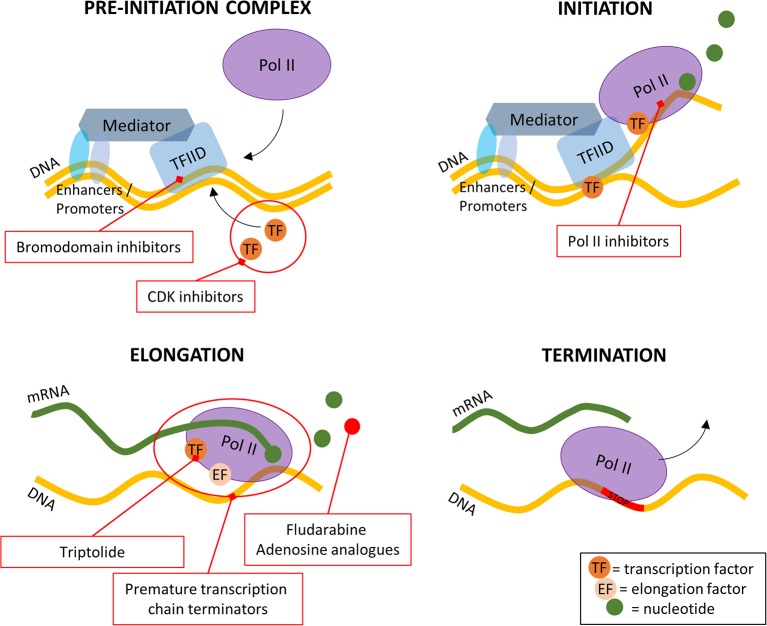
A simplified overview of the four stages of transcription and where the inhibitors are targeting. Pol II, RNA polymerase II; TFIID, transcription factor IID; CDK, cyclin dependent kinase.

Transcription is the process of mRNA synthesis. For this process to begin the chromatin must be accessible for the transcription machinery to assemble. The epigenetic state, that is the DNA methylation and histone modifications, of the chromatin determines this accessibility. Hence, one of the first levels of transcriptional regulation is the “open” or “closed” state of the promoter regions. The enzymes responsible for the addition or removal of these modifications are then the first drug targets in this process (Cheng et al., 2019). RNA transcription itself is a highly regulated multi-step process which involves many potential targets for drug development (Stellrecht and Chen, 2011; Villicaña et al., 2014). Mechanistically, transcription can be divided into four stages: (1) The formation of the pre-initiation complex, (2) transcription initiation, (3) transcription elongation, and (4) transcription termination (Figure 1). A more detailed biological overview of transcription can be found in other recent reviews (Cramer, 2019; Kujirai and Kurumizaka, 2019; Rodríguez-Enríquez et al., 2019; Babokhov et al., 2020).

Transcription of genes starts with the binding of transcription factor II D (TFIID) to the core promoter of the gene (Patel et al., 2020). There it starts the assembly of the large pre-initiation complex (PIC), which includes various transcription factors, cofactors, RNA polymerase II (Pol II), TFIIH and Mediator complexes. The xeroderma pigmentosum (XP)B and XPD subunits of TFIIH have helicase and ATPase activities which are needed for the opening of the promoter DNA double helix. The opening allows Pol II to start the mRNA transcription. The transcription initiation is finished after 25–30 nucleotides have been transcribed, and the process moves to the elongation phase. The transcription elongation consists of the release of the promoter, the binding of elongation factors and the hyperphosphorylation of the Pol II C-terminal domain (CTD) which all lead to enhanced stability of the transcription machinery (Stellrecht and Chen, 2011). The transcription terminates after the site of the polyadenylation has been transcribed and afterwards the mRNA is cleaved off the transcription machinery. Finally, the mRNA is modified by the cascade of proteins that take care of the 3′-end processing (Mandel et al., 2008).

Most small molecules used in targeted therapy affect transcription, at three of the four different steps of this process. On the pre-initiation complex, bromodomain, and extra-terminal motif inhibitors reversibly inhibit the ability of the bromodomain to bind to acetylated histones (Alqahtani et al., 2019), effectively slowing down the formation of the PIC (Figure 1). Still, at this first step, cyclin-dependent kinase (CDK) inhibitors have been researched and widely tested due to their role in the cell cycle (Blachly and Byrd, 2013). Their importance is not limited to cancer diseases (Malumbres and Barbacid, 2001, 2009) but also other diseases such as in HIV (Galons et al., 2013). At the second step of the transcription process, Pol II inhibitors block the formation of the Pol II—transcription factor complexes. The study of these inhibitors dates back to the late 80's and is still a subject of study today (Logan et al., 1989; Sharma et al., 2019) (Figure 1). The last step that can be acted upon at transcription level is the elongation step. There are two main methods of inhibiting this step: inhibition of transcription factors and usage of premature transcription chain terminators (Figure 1). After being considered undruggable for a long time, recent years have seen a growing number of transcription factor inhibitors and although the sheer number of known TF's makes it difficult to find a general acting drug, it also allows for a greater range of inhibitors to be developed (Bushweller, 2019). The transcription chain termination is naturally processed by Pols and to terminate it prematurely nucleoside analogs and/or inhibitors, such as fludarabine, are used.

In recent months, also antisense oligonucleotides (ASO)s have been detected to block transcriptional elongation (Poplawski et al., 2020) and termination (Lai et al., 2020; Lee and Mendell, 2020). Since these molecules have been much more studied in terms of translation, they will be discussed in more detail under translation inhibitors in section Antisense Oligonucleotides.

### Inhibitors of the Epigenetic Machinery

The epigenetic modifications including DNA methylation, histone modifications such as acetylation methylation, affect the transcription process without changes to the underlying DNA sequence (Smolle and Workman, 2013). Effectively epigenetic modifications determine the “openness” and structure of the chromatin, thereby affecting the DNA accessibility to the transcription machinery and consequently altering gene expression. Changes in the epigenetic landscape are commonly associated with cancer, and thus the enzymes responsible for the epigenetic changes can be inhibited to hinder the disease (Cheng et al., 2019). There are two recent and comprehensive reviews about epigenetic enzyme inhibitors and cancer, and thus this section will include only a short overview of the topic (Cheng et al., 2019; Roberti et al., 2019).

Different cancers have been associated with promoter hypermethylation in particular of tumor suppressor genes (Bouras et al., 2019). In addition, certain patterns of DNA methylation have been associated with drug resistance and the prediction of treatment efficacy (Wilting and Dannenberg, 2012). There have been hundreds of DNA methylation inhibitors in clinical trials against various cancers, and the research continues for important molecules such as azacytidine and decitabine (Cheng et al., 2019). Along with DNA methylating and demethylating enzymes, another main drug target of epigenetic machinery are the histone targeting enzymes, which include histone acetylases, deacetylases as well as histone methylases and demethylases (Zhao and Shilatifard, 2019). The effects of histone modifications in cancer have been reviewed by (Zhao and Shilatifard, 2019). In short, for histone acetylation related enzymes, there are tens of clinical trials in all three phases ongoing with for example vorinostat and panobinostat, which function as histone deacetylase inhibitors (Cheng et al., 2019). There are fewer clinical studies ongoing targeting histone methylases and demethylases, and none of these have advanced into phase III clinical trials (Cheng et al., 2019).

### RNA Polymerase Inhibitors

**Figure 2:**
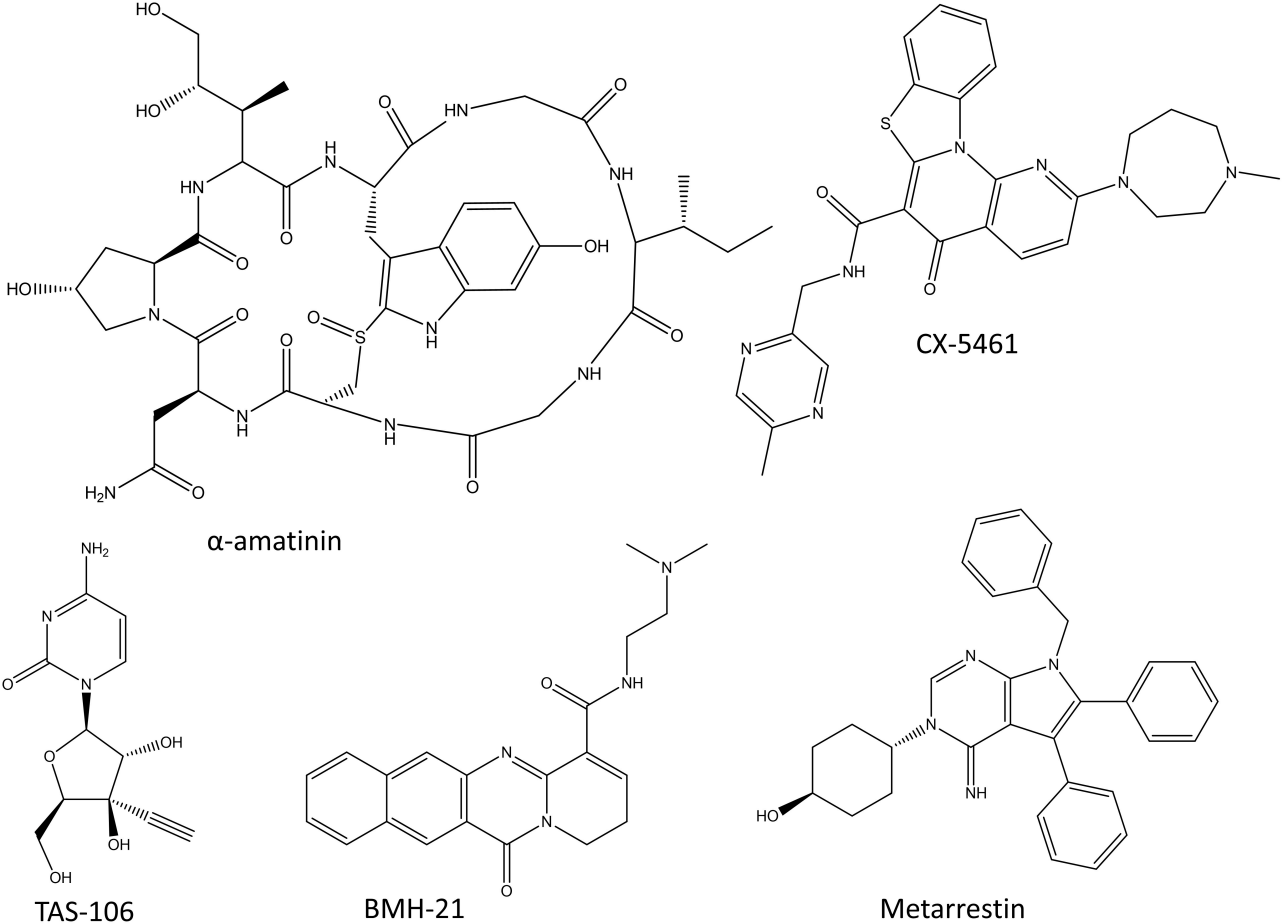
Chemical structures of RNA polymerase inhibitors.

RNA polymerases produce various RNA molecules. In humans, RNA polymerase I (Pol I) synthesizes the precursors of ribosomal (r)RNA, the main component of ribosomes; Pol II synthesizes precursors of mRNA and most of the snore (sn)RNA and micro (mi)RNAs; while RNA polymerase III synthesizes transfer (t)RNAs and other small RNAs. Cancer drugs or drug candidates have been developed to target all three human RNA polymerases. Some potent natural toxins, such as α-amatinin, inhibit RNA polymerases (Seifart and Sekeris, 1969). There have been efforts to convert α-amatinin into a cancer drug by combining it with antibodies for specificity. In addition to these conjugates, there are synthesized inhibitors of RNA polymerases, some of which have made it into clinical trials but unfortunately none of these RNA polymerase inhibitors have been approved for therapy as of yet (3/2020). Structures of the discussed RNA polymerase inhibitors are shown in Figure 2.

#### α-amatinin Conjugates

α-amatinin is one of the deadliest amatoxins produced by the death cap mushrooms (Lindell et al., 1970). It is a cyclic octapeptide (Figure 2) that inhibits both Pol II and Pol III by interacting with their bridge helices which slows down the translocation of the polymerase along the DNA strand and thus also the transcription process (Cochet-Meilhac and Chambon, 1974; Rudd and Luse, 1996). Since α-amatinin is very effective at killing both dividing and non-dividing cells, there have been efforts to conjugate it with antibodies to target it specifically to cancer cells (Pahl et al., 2018b). The preclinical studies have shown the effect of α-amatinin-antibody conjugates in pancreatic carcinomas and multiple myeloma cell lines (Moldenhauer et al., 2012; Pahl et al., 2018a). The more advanced amatinin-BCMA (B Cell Maturation Antigen; CD269) conjugate, HDP-101 is expected to enter clinical trials in the near future (Pahl et al., 2018a).

#### CX-5461

CX-5461 (Figure 2) is the first selective Pol I inhibitor that has finished phase I clinical trials with promising results in advanced hematological cancers (Khot et al., 2019). It prevents transcription initiation by inhibiting the binding of selectivity factor SL1 to the promoter region, and shows over 200-fold specificity toward Pol I over Pol II (Drygin et al., 2011; Haddach et al., 2012). The inhibition of Pol I transcription leads to cell-cycle arrest and cell death mediated by nucleolar stress response and DNA damage response mediated by p53 (Drygin et al., 2011; Bywater et al., 2012; Haddach et al., 2012). Even though the first clinical trials showed beneficial results and validated this relatively unexplored therapeutic approach, the development of CX-5461 into a commercial drug might be delayed due to the serious cutaneous side effects (Khot et al., 2019).

#### BMH-21

Small molecule BMH-21 (Figure 2) is a DNA intercalator which also inhibits Pol I in a manner which is not dependent on DNA damage (Peltonen et al., 2010; Colis et al., 2014). BMH-21 both inhibits Pol I and induces the degradation of the largest subunit of Pol I (Peltonen et al., 2014). The inhibition of Pol I by BMH-21 targets the transcription elongation phase and induces pausing in the transcription process (Wei et al., 2018). Despite the promising *in vitro* results, no clinical trials have been started with BMH-21.

#### TAS-106

TAS-106 (ECyd), 1-(3-C-ethynyl-β-D-ribo-pentofuranosyl)cytosine(3′-Cethynylcytidine; Figure 2), is a cytidine analog and a non-selective competitive inhibitor of all three RNA polymerases, thereby inhibiting RNA synthesis (Abdelrahim et al., 2013). It is a potent inhibitor of more than 40 kinds of cultured cancer cells and also human solid tumors xenografted into mice (Shimamoto et al., 2002; Abdelrahim et al., 2013). TAS-106 has been tested in multiple phase I and phase II clinical trials. The phase I studies have concluded that TAS-106 can be administered either as an infusion or as a bolus injection, and that the main dose-limiting adverse effect is its neurotoxicity (Friday et al., 2012; Hammond-Thelin et al., 2012; Naing et al., 2014). So far, the phase II clinical trials have not shown significant benefits for TAS-106 monotherapy and no new clinical trials have been started in the last years because of the lack of efficacy and the possibility of adverse effects (Tsao et al., 2013).

#### Metarrestin

One of the recent additions to the RNA polymerase inhibitors is metarrestin which functions by impairing Pol I-ribosomal DNA interaction and inhibiting the function of Pol I (Frankowski et al., 2018). It also inhibits the transcription of Pol I and disrupts the function of the perinuclear compartment which is a complex nuclear structure associated with metastatic cancer. At least some of the functions of metarrestin are mediated by its binding to the translation elongation factor eEF1A (Frankowski et al., 2018). Frankowski et al. (2018) tested the efficiency of metarrestin in multiple cell lines and pancreatic cancer xenograft mouse model with encouraging results.

### Transcriptional Complex Disruptors

**Figure 3:**
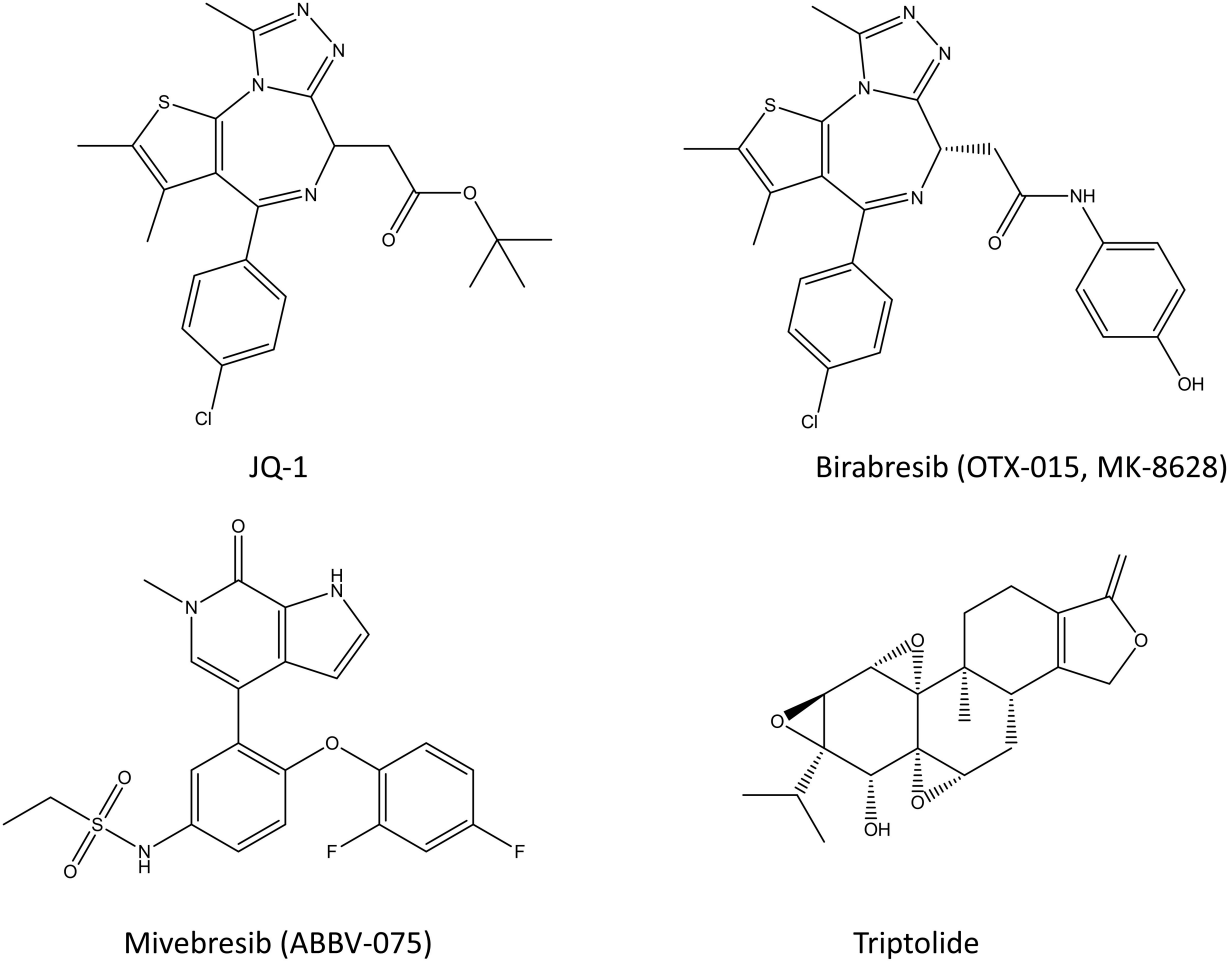
Chemical structures of various bromodomain inhibitors (JQ-1, birabresib, and miverisib) and triptolide.

Different complexes of proteins can be disrupted during transcription. The first complex to form is the pre-initiation complex which can be disturbed by bromodomain inhibitors. It is also possible to disturb the elongation process by preventing the binding of elongation factors to RNA polymerase, which is the mechanism of action of triptolide. The compounds included in this section are protein-protein interaction inhibitors, and their structures are shown in Figure 3.

#### Bromodomain Inhibitors

A short overview of the topic will be given here, since there are thorough reviews about bromodomain inhibitors from recent years (Pérez-Salvia and Esteller, 2016; Alqahtani et al., 2019; Letson and Padron, 2019). Bromodomain (BRD) inhibitors or BET (bromodomain and extraterminal domain) inhibitors prevent the interaction of bromodomain family proteins (BRD2, BRD3, BRD4, and BRDT) with acetylated histones and transcription factors (Filippakopoulos et al., 2010; Junwei and Vakoc, 2014; Fu et al., 2015). Since the bromodomain-containing proteins regulate gene expression through various processes including histone recognition and modification, chromatin remodeling, and regulation of the transcriptional machinery, BRD and BET inhibitors can be potent transcriptional inhibitors (Fujisawa and Filippakopoulos, 2017). In cancer, the acetylation state of histones and other proteins is altered, and BRDs promote the expression of many oncogenes, such as c-Myc and Bcl-2, and thus their inhibition provides a way to inhibit cancer cell growth (see also section Inhibitors of Transcription Factor Gene Expression) (Alqahtani et al., 2019). Most of the bromodomain inhibitors, such as JQ-1, compete for the acetylated lysine binding site and are thus competitive inhibitors (Alqahtani et al., 2019). However, some of its analogs have made it into the clinical trials (such as birabresib in leukemia and glioblastoma) (Alqahtani et al., 2019). The results of BET inhibitors as monotherapy have been suffering from resistance, lack of response and toxicity issues (Bolden et al., 2014; Letson and Padron, 2019), even though they were relatively effective in preclinical models of various cancers (Kharenko et al., 2016; Waring et al., 2016; Letson and Padron, 2019). This has sparked an interest in using BRD inhibitors in combination with other chemotherapeutic agents, which has shown promising results in animal models so far (Alqahtani et al., 2019). There are still dozens of on-going clinical trials for various BRD inhibitors in multiple different cancers with or without other chemotherapeutic agents. With more than 600 unique interaction partners in the cells, this family of proteins will continue to spark the curiosity of researchers for a long time.

#### Triptolide

Triptolide (Figure 3) is a diterpene triepoxide produced by thunder god vine, a plant regularly used in Chinese traditional medicine for rheumatoid arthritis (Su et al., 1990). In addition to inhibiting heat shock protein 70, it is an inhibitor of Pol I and Pol II which functions by blocking the transcription elongation process while binding to transcription factor TFIIH (Titov et al., 2011). It also facilitates degradation of the largest subunit of Pol II in a CDK7-dependent manner (Vispé et al., 2009; Manzo et al., 2012). Triptolide kills colorectal cancer cells *in vitro* and inhibits the growth of colorectal xenografts in a mouse model (Wang et al., 2009; Oliveira et al., 2015). Recently, Liang et al. tested triptolide in adenomatous polyposis coli (Apc) mutated mice where it effectively inhibited colorectal cancer proliferation (Liang et al., 2019b). Interestingly, they noted that triptolide also reduced Pol III mediated transcription by inhibiting TFIIIB formation at Pol III target genes. Specifically, it did so by blocking the interaction of TBP and Brf1 at Pol III promoters, thereby reducing tRNAs and 5S rRNA transcription. The inhibition of cancer growth both *in vitro* and *in vivo* makes triptolide a potential drug candidate for colorectal cancer. However, due to the toxicity of triptolide, only the prodrug disodium salt form of it, minnelide is being studied in human trials for pancreatic and liver cancers (Banerjee and Saluja, 2015).

### Premature Transcription Chain Terminators

**Figure 4:**
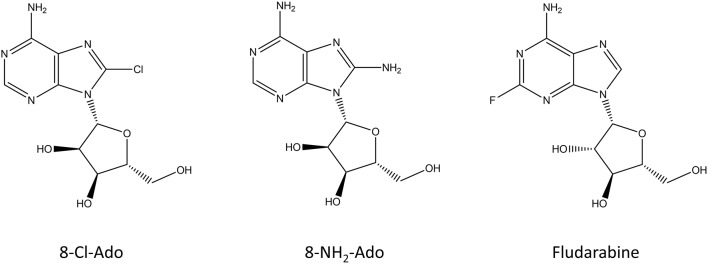
Chemical structure of the nucleoside analogs that function as premature transcription chain terminators.

Transcription is terminated when specific ending codons are reached on the DNA and after it the mRNA transcript unbinds from the RNA polymerase. The termination is coupled with the 3′-end processing which includes cleavage and addition of a poly-adenosine (A) chain (Cramer, 2004). This process can be disturbed by mimetics of adenosine or fludarabine (Figure 4).

#### Adenosine Analogs

Since transcription generally terminates when a poly-A chain is added to the mRNA transcript, modified adenosine analogs have been the focus of research as premature transcription chain terminators. 8-chloroadenosine (8-Cl-Ado) and 8-aminoadenosine (8-NH_2_-Ado) (Figure 4) can be incorporated to both the body and the poly(A) tail of the transcript, where they inhibit further synthesis of it (Gandhi et al., 2001). In addition to incorporation to mRNA, 8-Cl-Ado, and 8-NH_2_-Ado can be phosphorylated by adenosine kinase into corresponding ATP analogs which decreases the amount of available ATP in the cell (Frey and Gandhi, 2010). Interestingly, these compounds are not cytotoxic for non-transformed cells (Balakrishnan et al., 2005; Dennison et al., 2010). 8-Cl-Ado is in phase I clinical trials for acute myeloid leukemia and chronic lymphocytic leukemia (Stellrecht et al., 2017). Recently, 8-Cl-Ado showed a positive synergistic effect with another cancer drug in a mice xenograft model of acute myeloid leukemia (Buettner et al., 2019).

#### Fludarabine

Fludarabine (Figure 4) is a nucleoside analog which is used in the treatment of different leukemias and lymphomas (Gandhi and Plunkett, 2002). It was approved in 1991 by the FDA and it can be used either alone or in combination with other chemotherapeutics, such as cytarabine or mitoxantrone. Fludarabine is a prodrug that is converted into 9-beta-D-arabinosyl-2-fluoroadenine (F-ara-F) which can enter cells and accumulate as 5′-triphosphate-F-ara-ATP. The main functions of fludarabine are mediated through DNA incorporation or inhibition of DNA ligase and DNA primase (Stellrecht and Chen, 2011; Holzer et al., 2019). In addition to these, fludarabine can incorporate into RNA and inhibit the transcription process (Huang et al., 2000). The cytotoxic mechanism of fludarabine seems to be dependent on the cell type, and even a potassium channel was identified to be inhibited by it (Huang et al., 2000; de la Cruz et al., 2017).

### CDK Inhibitors

**Figure 5:**
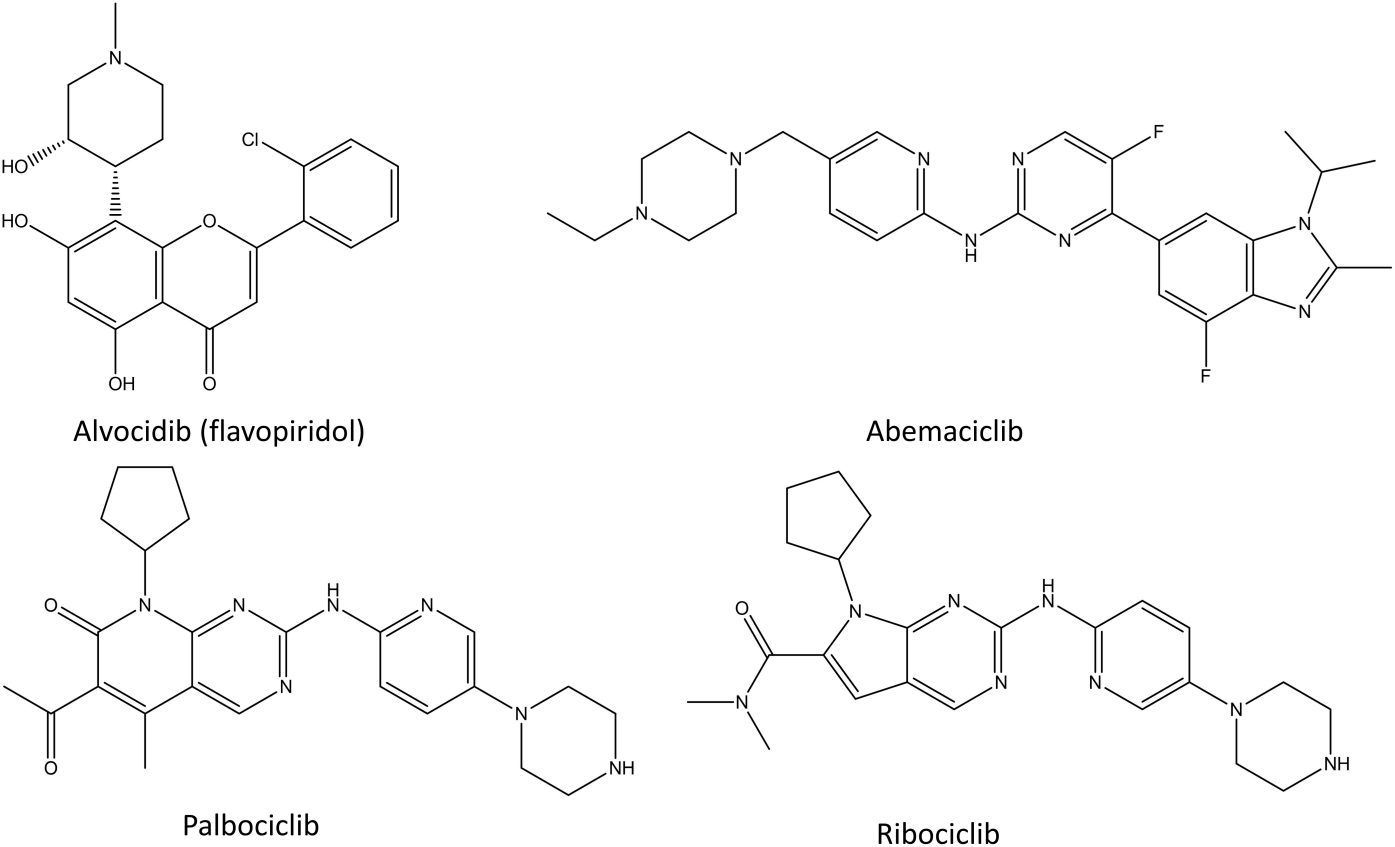
Chemical structures of selected CDK inhibitors.

Cyclin-dependent kinase (CDK) inhibitors are the newest class of transcription inhibitors that have gained approval by FDA and EMEA (Figure 5). Since 2015, palbociclib, ribociclib, and abemaciclib have been approved for the treatment of hormone receptor positive breast cancer. CDKs regulate the cell cycle by preventing the phosphorylation of transcription factors. In cancer their activity is many times distorted to ensure the proliferative state of the cancer cells. Since different CDKs control different parts of the cell cycle, it is beneficial to target them selectively instead of using pan-CDK inhibitors, such as alvocidib. All identified CDK inhibitors function as competitive inhibitors, binding to the ATP-binding site of these enzymes (Zeidner and Karp, 2015). The main issue in CDK inhibitors is the poor predictability of the patients response, that is if patients benefit from CDK inhibition and with what combination of other drugs (Asghar et al., 2015). Another issue is that they cannot be used in combination with many cytotoxic drugs or radiotherapy, since these act by stopping the cell cycle, whereas CDK inhibition therapies only work for cycling cells.

#### Alvocidib (Flavopiridol)

Alvocidib (formerly flavopiridol, Figure 5) is a semisynthetic flavonoid resembling rohitukine and it inhibits CDK1, CDK2, CDK4, CDK6, and CDK9. It was the first CDK inhibitor which reached clinical trials in 1998 (Senderowicz, 1999; Kelland, 2000). Since then more than 60 phase I and phase II clinical trials in various cancers have been conducted using it (Asghar et al., 2015). The broad target spectrum lead to promising *in vitro* results, but unfortunately the clinical tests showed only a little activity (Asghar et al., 2015). There are few positive results for leukemia and lymphoma, and new phase I and II clinical trials are continuously started for alvocidib (Byrd et al., 2007; Blum et al., 2011). Despite all the investments and thorough studies, alvocidib has not made it into phase III clinical trials, as of 2020. The main issues with alvocidib and other non-selective CDK inhibitors are the uncertainty of their mechanism of action, the problems in patient selection for clinical trials and the lack of a therapeutic window as a result of CDK inhibition in healthy cells (Asghar et al., 2015).

#### Palbociclib

Palbociclib (Figure 5) is a selective inhibitor of CDK4 and CDK6 and it was the first inhibitor of CDKs that was approved as a cancer therapy in combination with letrozole, an aromatase inhibitor (Lu, 2015; Turner et al., 2015; Finn et al., 2016). Targeting only these CDKs in tumors that have CDK4/CDK6 dysregulation, causes arrest of the cell cycle mediated by retinoblastoma 1 (Malumbres et al., 2004; Toogood et al., 2005). This results in lower levels of cyclins, nucleotide biosynthesis, DNA replication machinery and mitotic regulatory genes (Dean et al., 2010; Rivadeneira et al., 2010). Palbociclib is a product of multiple cycles of chemical screening and optimization which started from a set of pyrido[2,3-d]pyrimidin-7-one compounds with a 2-amino pyridine side chain at the C2 position that were showing specificity for CDK4/CDK6 over other CDKs (VanderWel et al., 2005). During the extensive clinical trials of palbociclib, its cytotoxic effect have been proven, and the main adverse effect has been neutropenia (Asghar et al., 2015). Neutropenia is a common adverse effect of chemotherapies, but in the case of palbociclib it is a rapidly reversible condition which can be avoided by intermittent dosage. The clinical tests also revealed that palbociclib has a beneficial effect in combination with hormone therapy in estrogen receptor (ER) positive breast cancer cell lines (Finn et al., 2009). Importantly, the inhibition of CDK4 and CDK6 showed activity in multiple ER positive cell lines that had developed resistance to ER antagonists (Miller et al., 2011; Thangavel et al., 2011). This lead to many phase II studies which confirmed the significant improvement in the median progression-free survival and granted palbociclib the Breakthrough Therapy designated from the FDA in 2013(Asghar et al., 2015).

#### Ribociclib

Ribociclib (Figure 5) was the second selective CDK4/CDK6 inhibitor to gain the market approval as a cancer therapy in combination with an aromatase inhibitor (Bartsch, 2017). It showed similar efficacy to palbociclib with a similar toxicity profile, with the addition of higher hepatotoxicity and rare cardiac QT time prolongation effects (Hortobagyi et al., 2016). Interestingly, whereas palbociclib is generally used for advanced states of cancer, ribociclib has also shown a positive effect in high-risk early-stage ER positive breast cancer (Prat et al., 2020).

#### Abemaciclib

Abemaciclib (Figure 5) was developed around the same time as palbociclib, and it gained FDA approval in 2017. Even though abemaciclib has similar mechanism of action and usage in cancer treatment for ER positive cancers as palbociclib and ribociclib, the main adverse effects of it are gastro-intestinal issues instead of neutropenia (Chen et al., 2016a; Sledge et al., 2017; Ettl, 2019). This is caused by non-specific inhibition of other kinases in addition to CDK4 and CDK6 and means that abemaciclib can be taken continuously unlike the other CDK4/6 inhibitors (Chen et al., 2016a).

## Targeting Translation

**Figure 6:**
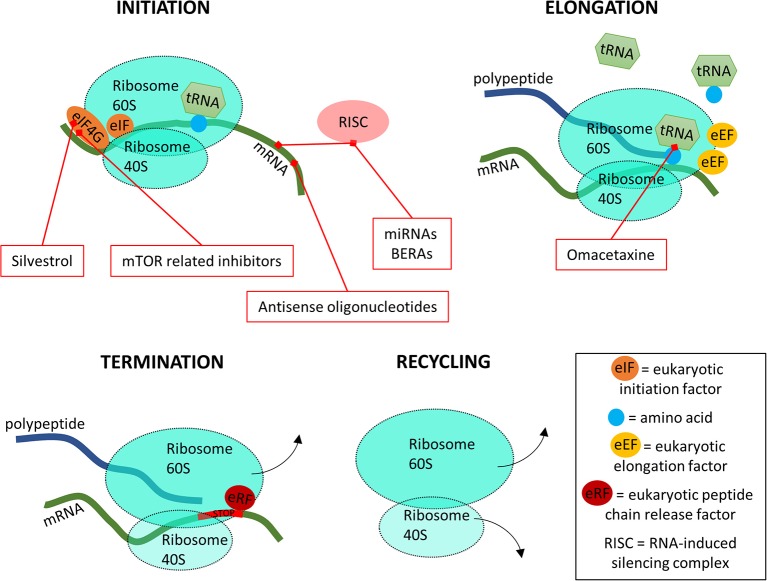
A simplified overview of the four stages of translation and where the inhibitors are targeting.

Translation is the process of polypeptide chain production according to the mRNA template (Figure 6). It includes dozens of druggable protein targets and consists of four stages: (1) translation initiation, (2) translation elongation, (3) translation termination and (4) recycling of the translation machinery (Roux and Topisirovic, 2018; Schuller and Green, 2018).

In the translation initiation phase, the 80S ribosome binds to the start of the mRNA after which tRNA carrying a methionine are able to bind to the starting codon AUG (Schuller and Green, 2018). The initiation phase is assisted by a wide variety of eukaryotic translation initiation factors (eIFs). For drug design, the most important eIF is eIF4G (Figure 6). During the elongation phase, the 80S ribosome moves along the mRNA template, binding new tRNA molecules with corresponding amino acids to synthesize the polypeptide chain. This process is coordinated by the eukaryotic elongation factors (eEFs). Once the 80S ribosome encounters a termination codon which is recognized by eukaryotic peptide chain release factors (eRFs), it releases from the mRNA and the polypeptide chain. Finally, the 80S ribosome complex separates into subunits 40 and 60S to begin a new round of translation. At the translation level, it has been shown that many signaling pathways are dysregulated in cancers (Wolfe et al., 2014; Faller et al., 2015). This association has been bringing translational control into the foreground of targeted cancer therapies (Vogel and Marcotte, 2012), where the spotlight was previously reserved for transcription level inhibitors. Here, the first step, initiation, is the most targeted by inhibitors, with the mammalian target of rapamycin (mTOR) inhibitors being in recent years one of the main targets of study (Figure 6) (Hua et al., 2019). Bioengineered non-coding RNA agents (BERAs) and antisense oligonucleotides, single-chain DNA that prevent translation by interacting with mRNA are also targeting this initial step (Duan and Yu, 2016; Jian et al., 2017). On the second step of translation, tRNA is targeted for inhibition, thus blocking the protein synthesis process. These inhibitors act by binding the free ribosome, interfering with the normal tRNA binding and thus blocking the elongation step (Figure 6) (Gandhi et al., 2014).

### mTOR Related Inhibitors

The mammalian (or mechanistic) target of rapamycin (mTOR) is a protein kinase which is the main component of mTOR complexes 1 and 2 (mTORC1 and mTORC2) which are one of the most important regulators of translation (Sabers et al., 1995). It functions as a serine/threonine protein kinase regulating many cellular processes, such as protein, lipid and nucleotide synthesis as well as nutrient sensing (Hsieh et al., 2012; Kim and Guan, 2019). The mTOR complexes affect translation by phosphorylation of multiple translation factors, including eIF4G (Iadevaia et al., 2012). The *MTOR* gene itself is often mutated in cancer (Grabiner et al., 2014). In addition, mTORC1 and mTORC2 affect oncogenic pathways, such as phosphatidylinositol-3-OH kinase (PI3K)-PKB/Akt pathway, and thus their signaling is frequently activated in cancer, for example in glioblastoma and follicular lymphoma (Kim and Guan, 2019). The readers interested in a more detailed overview of mTOR and its biology are referred to the recent and comprehensive review written by (Kim and Guan, 2019).

The inhibitors of mTOR complexes can be divided into three generations, first of which consist of rapamycin and its analogs termed rapalogs, which affect only specific parts of the mTOR complexes (Kim and Guan, 2019; Tian et al., 2019). The latter generation is termed ATP-competitive catalytic inhibitors or mTOR kinase inhibitors, and they target the catalytic activity of mTOR. The newest additions to the mTOR related drugs are called RapaLinks which consist of rapamycin linked with an mTOR kinase inhibitor, a combination of the previous two generations.

#### Rapamycin

**Figure 7:**
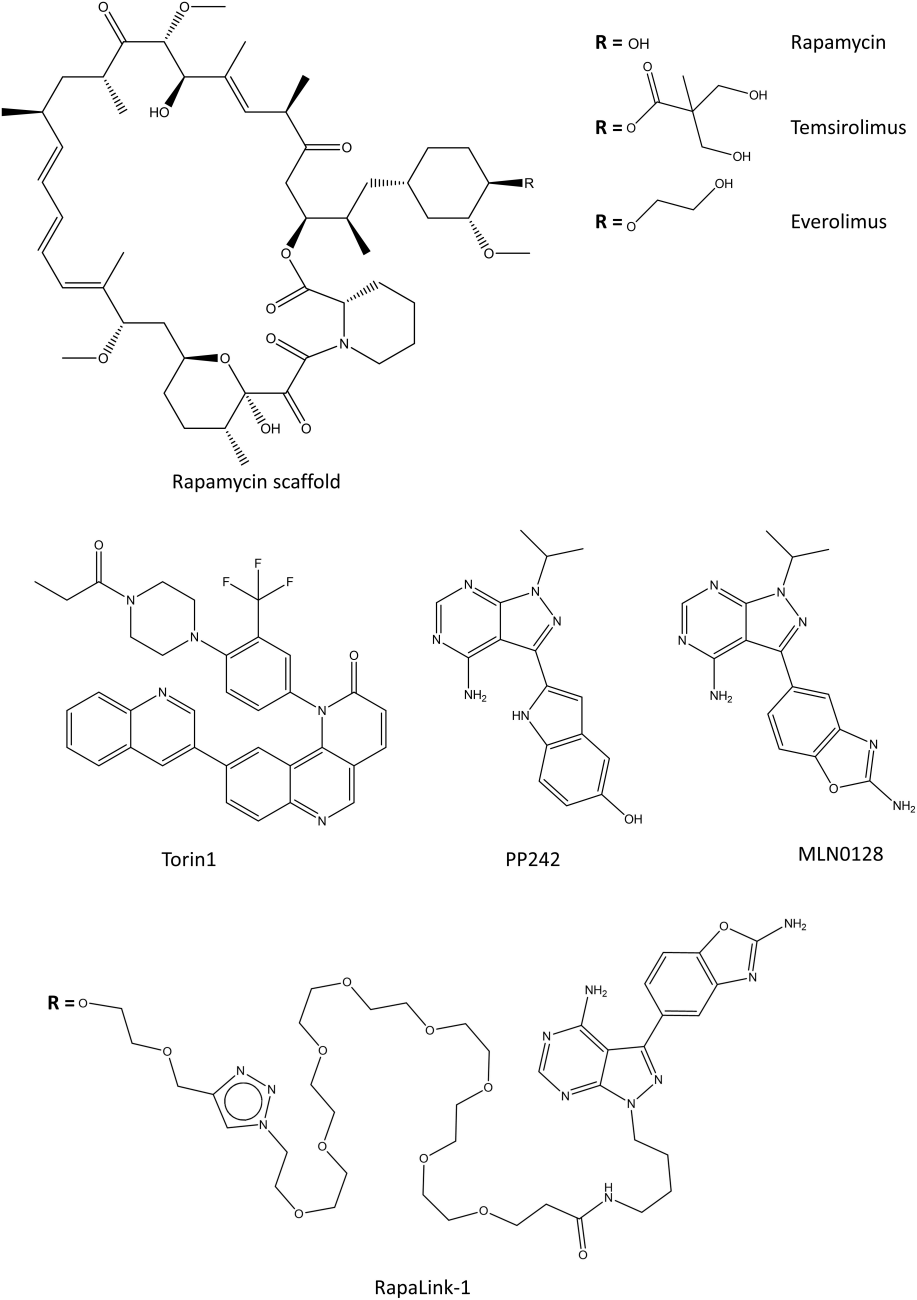
Selected chemical structures of inhibitors that target the mTOR complexes. Top contains the rapamycin, and its water-soluble analogs, rapalogs. In the middle there are some of the second generation of mTORC inhibitors which target the kinase activity. On the bottom, one of the third generation mTOR inhibitors is shown, which connects rapamycin scaffold with a second-generation kinase inhibitor.

Rapamycin (sirolimus, Figure 7) is an antifungal molecule produced by *Streptomyces hygroscopicus* and it was first discovered from a soil sample from the Easter Island (Rapa Nui) (Vézina et al., 1975). Structurally, rapamycin resembles the immunosuppressant tacrolimus, and it has similar immunosuppressive effect and mechanism of action via the inhibition of T- and B-cells (Sehgal, 2003). Because of these immunosuppressive properties rapamycin is one of approved drugs for the prophylaxis of renal transplantation (Pidala et al., 2012). It was later discovered that in addition to its antifungal and immunosuppressive properties, it is a potent inhibitor of many mammalian kinases (Chung et al., 1992; Kuo et al., 1992; Price et al., 1992; Sehgal, 2003). About 20 years after the discovery of rapamycin, its target was identified and aptly named, the mammalian target of rapamycin, mTOR (Sabers et al., 1995; Wiederrecht et al., 1995). Rapamycin functions by binding to 12-kDa FK506-binding protein (FKBP12) forming a complex which then allosterically inhibits mTORC1, but not mTORC2 (Li et al., 2014). Long-term rapamycin treatment can also affect mTORC2 signaling but the mechanism of this is not clear (Kim and Guan, 2019). Since mTORC1 complex is activated in numerous human cancers to keep the cancer cells proliferative and increase their nutrient uptake and energy metabolism, rapamycin impairs cancer metabolism and it has been thoroughly studied as a cancer drug (Li et al., 2014). However, the unmodified rapamycin has poor water solubility which leads to pharmacokinetic issues and facilitated the development of multiple analogs called rapalogs, the first generation mTOR inhibitors.

#### Rapalogs

Two water-soluble derivatives of rapamycin, temsirolimus, and everolimus (Figure 7), have been approved for the treatment of renal cancer carcinoma (Li et al., 2014). Everolimus is also approved for the treatment of progressive neuroendocrine tumors of pancreatic origin, and refractory mantle cell lymphoma in the EU. Even though many water soluble rapalogs were tested in cell and animal models, their effect in the clinic is generally only modest or weak (Kim and Guan, 2019). In some patients, rapalog usage inadvertently promotes cell survival by enhancing the PKB/Akt activity or by activating autophagy which promotes survival in oxygen-deprived microenvironments (Tabernero et al., 2008; Palm et al., 2015; Johnson and Tee, 2017). In some cases, these effects can be countered by combining rapalogs with an autophagy inhibitor hydroxychloroquine (Rangwala et al., 2014). Rapalogs function as allosteric inhibitors of mTORC1, and as such only prevent the phosphorylation of some of the mTORC1 substrates (Choo et al., 2008; Kang et al., 2013). This, in connection with the negative-feedback loop of PKB/Akt activation associated with the mTOR pathways is possibly the cause for the relatively poor clinical success of rapalogs.

#### mTOR Kinase Inhibitors

Torin1 and PP242 (Figure 7) were the first identified ATP-competitive mTOR inhibitors which block the kinase function of mTOR (Feldman et al., 2009; Thoreen et al., 2009). Later, Torin2 was characterized and tested alone and in combination with other kinase inhibitors against various cancer cell lines (Liu et al., 2013). Unlike rapamycin and rapalogs, mTOR kinase inhibitors directly inhibit the catalytic activity of both mTOR complexes. The mTOR kinase inhibitors display stronger inhibition of cancer cell proliferation than rapamycin due to their increased efficacy toward mTORC2. However, since mTOR signaling is essential for cell viability, blocking both mTOR complexes causes mTOR kinase inhibitors to have more severe side effects than rapamycin or rapalogs (Xie et al., 2016). The most advanced mTOR kinase inhibitor in clinical trials is a derivative of PP242, AZD2014, which is currently in phase II clinical trials for ER positive breast cancer (Guichard et al., 2015).

In some cases, the mTOR kinase inhibitors also inhibit PI3K which regulates mTOR activity, providing dual activity (Ballou and Lin, 2008). One such compound is wortmannin, a toxic steroidal furan produced by fungi (Brian et al., 1957). Wortmannin binds covalently to the ATP binding site of mTOR as well as PI3K. It is, however, toxic and instable in biological solutions which prevent its usage as a drug. Another dual kinase inhibitor, PI-103, is considered the first potent synthetic inhibitor of mTOR and it shows equal potency against PI3Ks (Ballou and Lin, 2008). It has been tested in mouse xenograft models of glioma (Fan et al., 2006). There are also other dual mTOR/PI3K inhibitors which have been tested in animals xenograft models (Koul et al., 2017; Shen et al., 2018; Yu et al., 2018; Zhao et al., 2019). There is a good therapeutic potential for dual mTOR/PI3K kinase inhibitors, and there are clinical trials ongoing for many of them, including LY3023414 and gedatolisib.

#### RapaLinks

The newest members of the mTOR targeting drugs consist of rapamycin linked with an mTOR kinase inhibitor, and they are aptly named RapaLinks (Rodrik-Outmezguine et al., 2016; Fan et al., 2017). In addition to having activity against many cancer cell lines, RapaLinks are effective against cancerous cells that are resistant to first or second generation mTOR inhibitors (Rodrik-Outmezguine et al., 2016). All published RapaLinks (1-3) have MLN0128 as the mTOR kinase inhibitor part, and they differ by the linker connecting the two-parts (Figure 7) (Rodrik-Outmezguine et al., 2016; Fan et al., 2017). MLN0128 by itself is not very effective *in vivo* due to its short residence time and its usage is limited by toxicity (Graham et al., 2018). Even though RapaLinks are large and contain a poorly water-soluble rapamycin, they can pass the blood-brain barrier and their efficacy has been shown in animal models of glioblastoma (Fan et al., 2017). If the *in vivo* results imply anything about clinical usability, we expect to see RapaLinks in clinical trials within the next few years.

#### Silvestrol

**Figure 8:**
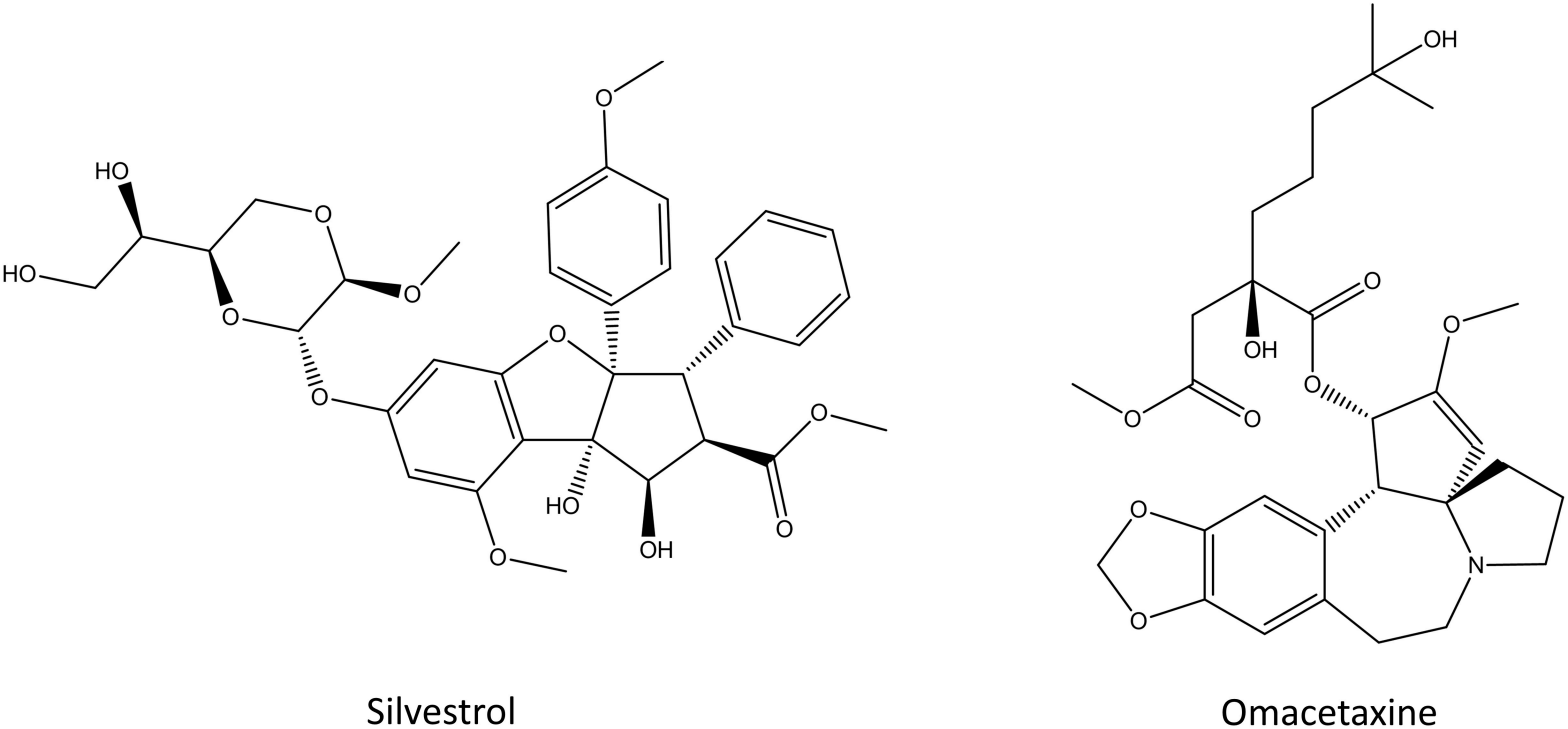
The chemical structures of silvestrol and omacetaxine.

Silvestrol (Figure 8) is a rocaglate derivative that can be isolated from the fruits and twigs of *Aglaia foveolate* (Pan et al., 2014). It is cytotoxic toward multiple cancer cell lines *in vitro* and it displays similar potency to paclitaxel or camptothecin (Hwang et al., 2004; Pan et al., 2014; Chen et al., 2016b). Silvestrol inhibits the translation initiation by binding to the initiation factor eIF-4A which prevents the ribosome loading onto the mRNA template (Cencic et al., 2009). This kills cells by inducing early autophagy and caspase-mediated apoptosis (Chen et al., 2016b). Silvestrol exhibits cytotoxic effects against different human cancer cell lines, such as melanoma, acute myelogenous leukemia, cervical cancer and oral carcinoma (Hwang et al., 2004; Lucas et al., 2009; Rodrigo et al., 2012; Kogure et al., 2013). Even though silvestrol is effectively cytotoxic against multiple cancer cell lines *in vitro*, only partial protein synthesis inhibition was observed in mice models of lymphoma (Bordeleau et al., 2008). The main issue with silvestrol and its analogs is that they upregulate multi-drug-resistant gene *ABCB1* and that they are substrates of p-glycoprotein, a well-known resistance-causing efflux transporter (Gupta et al., 2011). Despite the decade long research, silvestrol and its analogs remain at the preclinical drug research stage, and none of them has made it into clinical trials (Peters et al., 2018).

#### Omacetaxine

Omacetaxine, formerly known as homoharringtone (Figure 8), is a plant alkaloid from *Cephalotoxus fortune*. It was identified in 1970s as the inhibitor of the initial elongation step of translation (Huang, 1975; Fresno et al., 1977). Omacetaxine binds to the A-site cleft in the peptidyl-transferase site of the ribosome where it prevents the correct positioning of the incoming aminoacyl-tRNAs, thus functioning as a competitive inhibitor (Gürel et al., 2009). Because omacetaxine affects the elongation step, it is a more general translation inhibitor than other molecules that target translation initiation which inhibit only the translation of specific sequences (Wetzler and Segal, 2011). Treatment with omacetaxine leads to a rapid decrease in the number of proteins with short half-lives, including the oncogenic cyclin D1 and c-Myc (Robert et al., 2009). Omacetaxine was intensely studied after its discovery both *in vitro* and *in vivo* against chronic myeloid leukemia but after the approval of imatinib and other tyrosine kinase inhibitors, the scientific interest toward it dwindled (Wetzler and Segal, 2011). Recently, new clinical studies around omacetaxine have been started due to its synergistic effect with tyrosine kinase inhibitors, especially in the treatment of cancers with mutations in the tyrosine kinase genes (Marin et al., 2005; Cortes et al., 2012; Rosshandler et al., 2016; Maiti et al., 2017). Omacetaxine is approved by the FDA for the treatment of chronic myeloid leukemia if the disease does not respond to two or more tyrosine kinase inhibitors (Cortes et al., 2012, 2013; Rosshandler et al., 2016). This way, omacetaxine can help patients who suffer from lack of effect of those drugs, intolerance or drug-drug interactions. The most common adverse effects of omacetaxine are myelosuppression and thrombocytopenia which are observed in almost all patients but they can be managed with supportive care, dose delays and reduction in the number of days that omacetaxine is administered (Rosshandler et al., 2016).

### Antisense Oligonucleotides

Antisense oligonucleotides (abbreviated either ASO or AON) are short (13–25 nucleotides), single stranded DNA molecules that are complementary to target RNA sequences. In the cell, these oligonucleotides can hybridize to target RNA sequences, including mRNA and non-coding (nc)RNA to inhibit their expression and thereby regulate the availability of specific proteins. Different chemical modifications of synthesized oligonucleotides have been made to increase their nuclease stability, decrease non-specific effects and to improve their cellular uptake (Karaki et al., 2019; Yin and Rogge, 2019). These include phosphorothioates (PS) and derivatives of, phosphorodiamidate morphilino oligomers, peptide nucleic acids (PNAs), and chimeric 2′-O-methyl/phosphorothioate ASOs (Crooke, 2017). In addition to increased stability, some of these modifications have also enabled the oligomer binding to double-stranded DNA and have altered mechanisms of action.

ASOs have different mechanisms of actions including enzyme-mediated target RNA degradation, steric-hindrance of translation, as well as modulation of splicing and transcription (MacLeod and Crooke, 2017). Historically, the first mechanism of ASOs identified was RNase H-dependent degradation, which entails the hydrolysis of the RNA strand in a RNA/DNA duplex (Crooke, 2017). This is efficiently mediated by the ubiquitous RNase H and has the advantage that the oligonucleotide can be targeted to any part of the RNA molecule. However, problems of specificity due to activation following partial hybridization have been observed and pose a concern. Modified oligomers deviate from RNase H-induced cleavage and can inhibit protein expression via other mRNA quality control decay pathways. These include the non-sense-mediated decay (NMD) (Ward et al., 2014; Nomakuchi et al., 2016) and the no-go decay (NGD) (Liang et al., 2019a) which is triggered by ribosome stalling due to obstacles and collision of multiple ribosomes on the mRNA (Harigaya and Parker, 2010). Oligonucleotides targeted to either the 5′ or 3′ splice sites can interfere with splicing (Havens and Hastings, 2016; Singh et al., 2018). Interestingly, this can be used either to block mature protein expression or to correct aberrant splicing thereby restoring the protein function. Furthermore, oligonucleotides can result in steric hindrance of translation by preventing ribosome binding when targeted near the translation initiation codon (Chery, 2016; Goyal and Narayanaswami, 2018). Alternatively, ASOs targeted to the 5′UTR can inhibit translation by preventing 5′ cap formation.

In recent years with the growing identification and appreciation of the role of non-coding RNAs in transcription and gene regulation, ASO targeting non-coding RNAs have now also been implicated in modulation of transcription. In addition, to indirect transcription effects of ASOs due to RNAse H-reduction of specific non-coding RNAs, such as enhancer, promoter or long-noncoding RNAs (Li and Chen, 2013), in recent months, ASOs have also been implicated in blocking transcriptional elongation (Poplawski et al., 2020) and termination (Lai et al., 2020; Lee and Mendell, 2020). Specifically, ASO mediated degradation of nascent RNA also inadvertently resulted in premature transcription termination which can be avoided by targeting the ASO to the 3′ end of the transcripts. It is likely, that new effects on transcription will be identified with increasing use of ASOs in the non-coding RNA field and better understanding of their functions.

The power of ASOs as therapeutic agents has long been realized with FDA approval of the first ASO already in 1998 and 5 approved to date for nervous muscular or familial metabolic diseases (Stein and Castanotto, 2017; Yamakawa et al., 2019). However, antisense therapy for cancer treatment has lagged behind and to date there are no approved ASO therapeutic for cancer. Nevertheless, there are many ongoing clinical trials using ASOs targeting primarily cell proliferation and signaling as well as cancer stroma and resistance to chemotherapy. The most advanced ASO in the clinical trials is trabedersen which is in phase II trials. Ongoing clinical trials with ASOs targeting Bcl-2 (NCT04072458), Grb2 (NCT04196257), and androgen receptor (AR; NCT03300505) as well as future mRNA (Laikova et al., 2019) and non-coding RNA targets (Slack and Chinnaiyan, 2019) for the treatment of various solid tumors will in time tell of the efficacy of ASOs for cancer treatment.

#### Trabedersen (AP 12009)

Trabedersen (AP 12009) with the sequence 5′-CGGCATGTCTATTTTGTA-3′ is the most advanced ASO in clinical trials against cancer. Trabedersen is directed against TGF-β2 (transforming growth factor beta 2), which is overexpressed in cancer in particular in glioma and is associated with tumor progression (Kjellman et al., 2000). In a randomized, dose-finding Phase IIb clinical trial, AP 12009 at 10 μM was found to be safe and to have superior efficacy over a higher dose of AP 12009 or chemotherapy treatment (Bogdahn et al., 2011). With these encouraging results a multicenter phase III trial was initiated, however it was discontinued due to patient recruitment failure (NCT00761280). AP 12009 has also been tested for the treatment of patients with advanced pancreatic carcinoma, metastasizing melanoma, or metastatic colorectal carcinoma and a phase II trial demonstrated encouraging survival results (Stauder et al., 2004; Oettle et al., 2011).

#### AZD4785

Another ASO that has shown promising results was AZD4785. AZD4785 is a cET-ASO targeting KRAS (Ross et al., 2017), an oncogene that is often mutated in association with cancer. AZD4785 was previously shown to efficiently deplete KRAS and was associated with an antitumor effect in mice (Ross et al., 2017; Sacco et al., 2019). In addition, cellular trafficking and localization of AZD4785 across different tumor cell lines have been characterized and was found to vary (Linnane et al., 2019). Following completion of a phase I trial the molecule was demonstrated to be safe and well-tolerated, but it was discontinued by AstraZeneca because of its insufficient efficacy possibly due to targeting both mutant and wild-type KRAS mRNA (Yang et al., 2019).

### RNA Interference-Based Inhibitors

Following the seminal discovery that double stranded RNAs (dsRNAs) could cause post-transcriptional gene silencing (PTGS) in *C. elegans* (Fire et al., 1998), it did not last many years until RNA-based therapeutic inhibitors were in clinical trials (Whelan, 2005) and in 2018 patirisan received FDA approval as the first RNA based drug for the treatment of polyneuropathy. RNA interference (RNAi) as an inherent cellular process of RNA-mediated suppression of gene expression; it achieves this by inhibition of mRNA translation or targeting mRNA for degradation or sequestration (reviewed in detail in Jinek and Doudna, 2009; Setten et al., 2019). The short interfering (si)RNA required for activation of the RNAi pathway, can be derived exogenously from synthetic dsRNA or small hairpin (sh)RNA, in parallel endogenous miRNA can be generated following processing of transcribed pri-micro (mi)RNA. Whereas, the miRNA biogenesis pathway involves cleavage of pri-miRNA in the nuclear microprocessor complex and then with Dicer complexed to TAR RNA binding protein (TRBP) in the cytoplasm, shorter dsRNA can bypass this and is similarly but directly incorporated into the RNA-induced silencing complex (RISC) (Ha and Kim, 2014; Setten et al., 2019). In this complex, Argonaut 2 (AGO2) removes the passenger (sense) strand and the guide (antisense) strand is maintained, normally due to higher 5′ stability, in the mature and active RISC. In the case of siRNA, the guide RNA is then able to bind with perfect complementarity to target mRNA within the coding region, and in so doing results in mRNA cleavage by the AGO endonucleases. In contrast, miRNA that can bind to several mRNAs and often have their target site in the 3′UTR of mRNA, tend to do so with only partial complementarity. The partial base pairing compromises the AGO slicer catalytic activity and instead results in either translation repression or degradation of mRNA. In both cases small RNA result in suppression of mRNA and subsequently the matching protein.

Activation of RNAi and the use of siRNA for therapeutic means have the appeal of small molecules but have the added value of specificity and the flexibility of target selection. For these reasons some siRNA molecules were already in clinical trials within 10 years of their discovery. However, early clinical trials with siRNAs failed, some of which due to non-specific activation of the innate immunity. This motivated new discoveries, such as siRNA activation of Toll-like 3 pathway (Kleinman et al., 2008) and it necessitated further development of RNAi drugs in the form of chemical modifications. For a comprehensive review see (Khvorova and Watts, 2017). These modifications not only served to increase safety by avoiding dsRNA activation of the immune response, but they also increased the potency and stability of dsRNA by increasing their resistance to endonucleases, as well as in some instances facilitating antisense strand selectivity (Zuckerman and Davis, 2015). In addition to chemical modifications of dsRNA, progress in targeting and packaging of these for improved delivery of RNAi drugs was also necessary (Pecot et al., 2011; Juliano, 2016; Dowdy, 2017). Successful packaging of dsRNA was achieved in nanoparticles, polymers and dendrimers to name a few, and targeting has been accomplished with aptamers, antibodies, peptides and small molecules (Zhou and Rossi, 2017; Springer and Dowdy, 2018).

In addition to siRNA-based therapies, miRNA therapeutics have also been in development, in this case either as endogenous miRNA replacement or inhibition strategies (Hanna et al., 2019; Takahashi et al., 2019). In cases where antagonism of the miRNA is desired a synthetic, single-stranded RNA is introduced to target the miRNA for degradation and thereby inhibit its activity and disease progression. In contrast, miRNA replacement strategy is intended for reactivation of a particular miRNA pathway and the associated translation inhibition by replenishment of a specific miRNA in the form of miRNA mimics. Both of these strategies can be useful for cancer treatment, either in inhibiting oncogenes or gene products facilitating cancer growth or to reactivate miRNAs that are downregulated in tumors (Van Roosbroeck and Calin, 2017; Takahashi et al., 2019).

RNA therapeutic avenues are likely to extend in the future, as we are not limited to RNAi mechanisms in the cytoplasm but dsRNAs can also act in the nucleus to cause transcriptional gene silencing (TGS) via modification of epigenetic marks (Castel and Martienssen, 2013; Martienssen and Moazed, 2015). In addition, siRNA targeting gene promoters can also cause transcriptional activation (Laham-Karam et al., 2018), these transcription regulating small RNA can expand the repertoire of the RNA therapeutics and are likely to reach the clinics in future.

#### G12D KRAS -Targeted siRNA

Today, many RNAi drugs for cancer therapy are in clinical trials. One of these is siG12D LODER, which is a siRNA against the cancer-associated mutant KRAS (siG12D) encapsulated in a miniature biodegradable implant, Local Drug EluteR (LODER) (Khvalevsky et al., 2013). LODER is a polymeric matrix of poly(lactic-co-glycolic) acid that facilitates prolonged delivery of siRNA and has been tested for the treatment of pancreatic cancer. Following preclinical safety and toxicity assessment (Ramot et al., 2016), the siG12D LODER, was evaluated in phase 1/2a clinical trial (NCT01188785) in association with chemotherapy for patients with non-operable locally advanced pancreatic cancer (Golan et al., 2015). The RNAi drug was found to be safe and well-tolerated despite some adverse reactions and importantly demonstrated anticancer effects. It has now proceeded to Phase II trials.

#### EphA2-Targeted siRNA

Another non-liposomal siRNA delivery system undergoing testing is 1,2-Dioleoyl-sn-Glycero-3-Phosphatidylcholine (DOPC) EphA2-targeted siRNA (Landen et al., 2005). EphA2 is a tyrosine kinase receptor that normally functions in neuronal development but its overexpression has been observed in human cancers and decreased expression can reduce tumorigenicity (Ieguchi and Maru, 2019). Since DOPC is a neutral lipid complex it is expected to have lower toxicity compared to charged liposomes and as such was tested with a siRNA targeting EphA2 (Wagner et al., 2017). In these safety studies in murine and primates EPHRNA was found to be well-tolerated at different doses.

#### miRNA-34a Prodrugs and Mimics

miRNA-34a is one of the endogenous miRNAs that have been of interest as cancer drug design targets (Zhang et al., 2019a). Both prodrugs of it and mimics have been tested for cancer treatment (Zhao et al., 2015; Beg et al., 2017). In a multicenter Phase I clinical trial using the synthetic miRNA34a mimic, MRX34, patients with refractory advanced solid tumors were treated with liposome encapsulated MRX34 at escalating doses (Beg et al., 2017). However, despite evidence of antitumor activity in some patients this trial was terminated due to serious adverse events (https://clinicaltrials.gov/ct2/show/NCT01829971). Although miRNA targeted therapy remains appealing the feasibility of such therapy is still to be proven.

### BERAs

Although different chemical modification of synthetic RNA molecules intended for RNAi therapeutics have increased stability and demonstrated favorable pharmacokinetics properties, these Chemo-engineered RNAs are different from naturally transcribed RNA molecules in living cells, which are largely unmodified. This difference affects the structure, properties, and possibly the activity and immunogenicity of these molecules (reviewed in Yu et al., 2019). Also, effort has been made to bioengineer RNA molecules in living cells, including in bacteria and yeast (Huang et al., 2013; Chen et al., 2015; Pereira et al., 2017b; Ho et al., 2018; Kaur et al., 2018; Duman-Scheel, 2019). These bioengineered RNA agents (BERA) can be produced in large scale and carry no or minimal posttranscriptional modifications (Li et al., 2015; Wang et al., 2015). Different strategies for producing BERA have been tested, including strategies using RNA-binding proteins such as viral p19, inclusion into tRNA or 5S rRNA scaffolds and tRNA/pre-miRNA chimeras. The last of these has proven to be the most versatile platform as siRNA, miRNAs, and RNA aptamers of different sizes and forms have been produced at large scale with high yields (Duan and Yu, 2016; Yu et al., 2019).

Importantly, the BERAs produced have demonstrated biological activity in cells and in animal models. Examples of these tested for tumor treatment, are miR-34a prodrugs. In a xenograft mouse model using NSCLC A549 carcinoma cells, a bioengineered miR-34a prodrug in the form of a tRNA/mir-34a chimera, mediated tumor suppression following intra-tumor injection (Wang et al., 2015). Likewise, systemic delivery of an improved miR-34a-5p prodrug significantly decreased metastatic lung xenograft tumor growth in mice (Ho et al., 2018). In addition, other formulation of miR-34a prodrugs have resulted in similar findings in orthotopic osteosarcoma xenograft tumor mouse model (Zhao et al., 2016) and in combination therapy to reduce pulmonary metastases and osteosarcoma progression (Jian et al., 2017). In both these studies, it was also shown that the therapeutic doses of mir-34a prodrug were well-tolerated as indicated in blood chemistry profiles monitoring for hepatic and renal toxicities. Recently, another miRNA prodrug was investigated targeting pancreatic cancer (Li et al., 2015; Tu et al., 2019). A bioengineered miR-1291 was tested alone or in combination with chemotherapy treatment in PANC-1 xenograft and pancreatic cancer patients derived xenograft (PDX) mouse models and was found to be effective in reducing tumor growth and was well-tolerated (Tu et al., 2019).

Vulnerability of naked BERAs to RNAse-degradation in blood necessitates additional formulation for BERA to protect the RNA molecules. This can be done by cationic lipids, polymers, and peptides (Kim et al., 2016). Specifically, polyethylenimine (PEI)-based polyplexes (complexes of nucleotides and polycations) have facilitated efficient delivery in tumor models (Zhao et al., 2016; Tu et al., 2019). However due to potential toxicity of polyplexes (Lv et al., 2006), formulation based on lipidation of these has been recently tested for delivery of tumor associated miRNA (Zhang et al., 2018; Jilek et al., 2019). Increased serum stability of these BERAs as well as improved delivery, therapeutic effectiveness and survival of tumor bearing mice were observed. These positive results encourage the further development of BERA for tumor therapy.

## Targeting Oncogenes

Oncogenes are genes that can cause cancer once mutated or when expressed at high levels (Croce, 2008). Many oncogenic pathways lead to altered transcription or translation of various proteins. In order to keep the topic of this review we will focus on two oncogenic targets that are involved with transcription and translation; transcription factors and KRAS. Both of these oncogenes were previously thought to be undruggable but nevertheless, a few inhibitors for both of them have been published in the last few years. The readers interested in the drugs designed for oncogenic kinases or other oncogenic pathways are referred to other reviews (Bhullar et al., 2018; Solassol et al., 2019; Tang and Zhao, 2019; Wang et al., 2019; Zhang et al., 2019b).

### Transcription Factors

**Figure 9:**
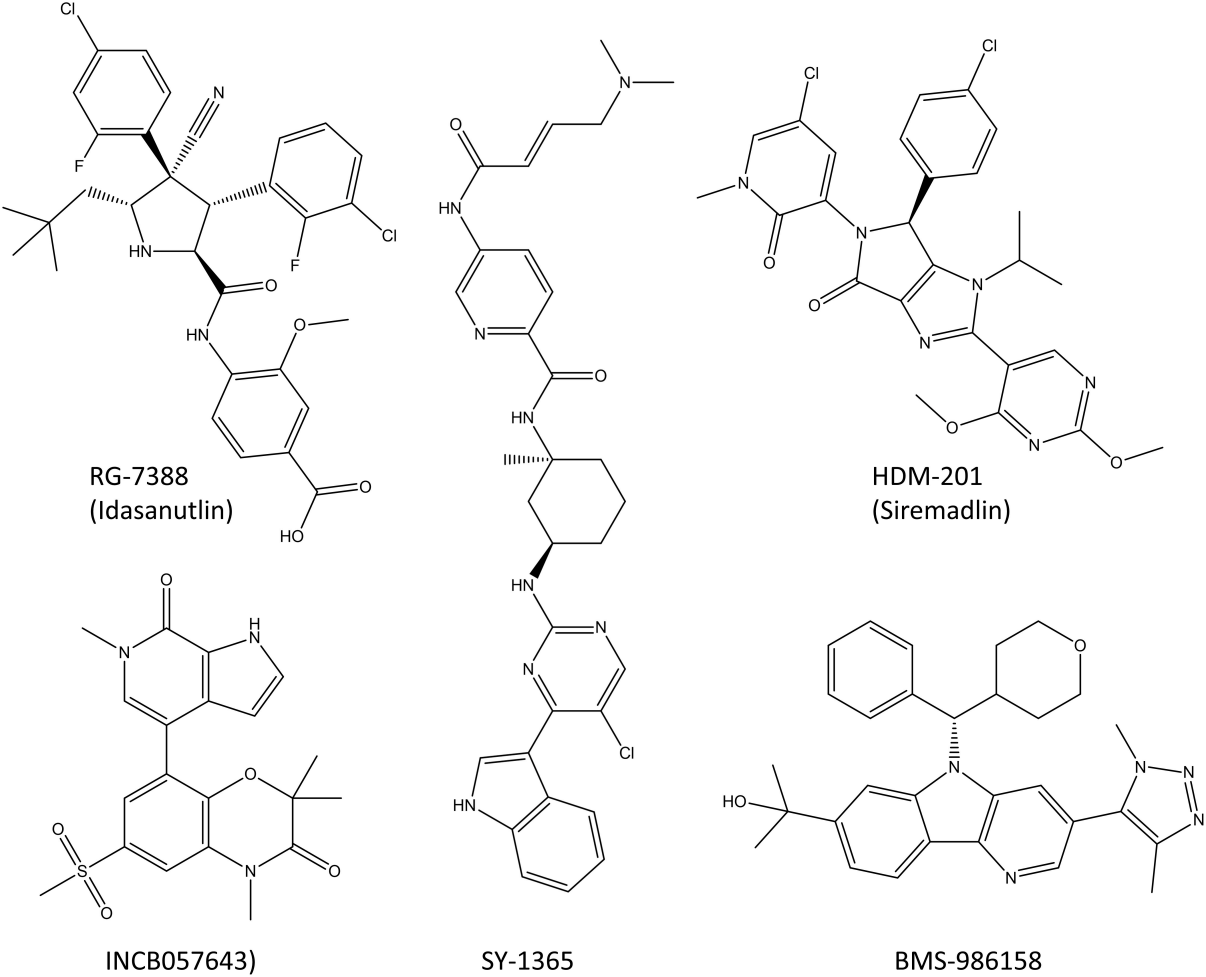
Molecules that target transcription factors and are in clinical trials or close to starting them. RG-7388 and HDM-201 are PPI inhibitors, SY-1365 is a selective CDK7-inhibitor and INCB057643 and BMS-986158 are BET inhibitors that block the transcription of many transcription factors, including c-Myc.

The idea of targeting transcription factors in cancer has been around about 20 years (Darnell, 2002). Transcription factors can drive oncogenesis as fusion proteins or by chromosomal translocation events (Bushweller, 2019). The DNA binding site of transcription factors with its positively charged environment is a difficult target for developing small-molecule inhibitors, and thus most of the recent efforts have been aimed for the protein-protein interaction (PPI) inhibition, such as RG-7388 (Arkin et al., 2014; Zhong et al., 2019). Transcription factors can be directly targeted by disrupting their transcription or translation, stabilizing their auto-inhibitory states, inducing covalent modifications with cysteine bridges or changing their post-translational modifications (Bushweller, 2019). Here we will shortly present the most advanced molecules that target transcription factors and are in or close to starting clinical trials (Figure 9). More detailed insights of targeting transcription factors in cancer can be found in the excellent review by (Bushweller, 2019).

#### Protein-Protein Interaction Inhibitors

So far, four different PPI inhibitors that target transcription factors have made it into the clinical trials or are very close to starting them (Bushweller, 2019). Two of these, RG-7388 (idasanutlin) and HDM201 (siremadlin; Figure 9), prevent MDM2 binding to p53 which prevents the degradation of p53 and increases its cellular levels leading to increased cell death (Ding et al., 2013; Furet et al., 2016; Skalniak et al., 2018). RG7388 and HDM201 have multiple phase I clinical trials ongoing both against solid tumors such as melanoma as well as hematological malignancies such as leukemia. The clinical trials in leukemia are planned for KO-539 and SNDX-5613 which target the mixed lineage leukemia (MLL) transcription factor and inhibit its binding to menin which prevents this fusion protein from activating genes driving leukemia. Unfortunately, the structures of these inhibitors have not been made public yet.

#### Inhibitors of Transcription Factor Gene Expression

There is one CDK-inhibitor (SY-1365) in phase I clinical trial for ovarian and breast cancer (Hu et al., 2019). The inhibition with SY-1365 leads to decreased levels of multiple oncogenic transcription factors and it exhibits the inhibitory effects on multiple cancer cell lines at nanomolar level. In addition, mouse xenograft studies showed modest antitumor activity in both AML as well as ovarian cancer, and a synergistic effect with venetoclax (Hu et al., 2019).

Two BET inhibitors are in clinical trials that target specifically the transcription of transcription factors, INCB057643 and BMS-986158 (Forero-Torres et al., 2017; Gavai et al., 2018). Both inhibitors lower the expression levels of the c-Myc oncogene and the proliferation rates of multiple cancer cell lines, and in animal models they display suitable properties for oral dosing in humans. The clinical trials are ongoing for advanced cancers, both solid tumors and leukemias.

### RAS Inhibitors

Drugs affecting transcription and translation are difficult to develop. This statement has been a harsh reality for those who have aimed to design oncogene RAS inhibitors, especially inhibitors of KRAS. The RAS GTPase family includes HRAS (Harvey rat sarcoma viral oncogene homolog), KRAS (Kristen rat sarcoma viral oncogene homolog), KRAS split variant KRASB (KRASA is same as KRAS) and NRAS (neuroblastoma RAS viral oncogene homolog). As RAS proteins are the most commonly mutated proteins in cancer and at the same time are part of the signaling cascade from the EGFR receptor, these have been a natural target for drug discovery. After more than 30 years of research, we now have the first compounds in clinical trials targeting mutated RAS protein, namely G12C KRAS. The difficulty of this process has been aptly stated in a recent editorial in the British Journal of Cancer “To put this development into context, KRAS was first described in 1983 and it has taken 35 years to reach this point, whereas identification of oncogenic BRAF mutations in 2002 was followed by an effective targeted drug in 2009” (Lindsay and Blackhall, 2019).

RAS proteins are small GTPases, thus removing the gamma-phosphate of GTP to produce GDP. The biological function is related to conformational cycle between GTP-bound active RAS and GDP-bound inactive RAS. The GTP-bound RAS can adopt a conformation able to bind with downstream signaling kinases (like PI3K or RAF) and this allows further activation of kinase pathways. Those interested to know more about RAS protein structure and function are advised to look at recently published review (Pantsar, 2020) and another review about early drug discovery work on RAS by Ostrem and Shokat (2016). In this part of our review we will concentrate on the recent works of KRAS G12C targeting covalent inhibitors as those are the most promising KRAS inhibitors currently known. The KRAS -targeting siRNA was discussed in section G12D KRAS -Targeted siRNA.

As stated KRAS is cycling between inactive GDT-bound and active GTP-bound stated. Direct targeting of GTP-binding pocket is not a realistic option as GTP has a high 0.5 mM concentration near the intracellular site where RAS proteins are located (Traut, 1994) and at the same time low femtomolar binding affinity (John et al., 1993; Ford et al., 2009). As there are no other clear druggable pockets in RAS direct targeting seemed to be an impossible mission. The problem was partially solved by the seminal work of Shokat lab which demonstrated that covalent interaction targeting mutated G12C residues is able to deliver *in vivo* relevant inhibition of RAS activation (Ostrem et al., 2013). The initial compounds presented were developed by disulphide-fragment based screening using tethering compounds. After early hit-optimization guided by X-ray crystallography an optimized G12C targeting covalent inhibitor was presented. In the optimized compounds disulfides were replaced by different electrophilic warheads and especially acrylamides were used. Upon covalent interaction G12C position compounds were binding previously unknown allosteric pocket. Even more important is the finding that these G12C inhibitors are RAS-GDP specific. This effect will undoubtedly explain the positive clinical outcome of two later-developed G12C inhibitors, namely AMG 520 and MRTX849 (Canon et al., 2019; Hallin et al., 2020). Both AMG 520 and MRTX849 seem to give better results with combination of either upstream or downstream kinase inhibitors. This is not surprising as such, but more surprising is a very recent report concerning the rapid non-uniform adaptation to KRAS G12C inhibition (Xue et al., 2020). According to Xue et al. (2020) G12C inhibitors targeting the RAS-GDP state promote a feedback mechanism in which KRAS G12C is maintained in active state by EGFR and Aurora kinase A (AURKA) signaling. If this finding is validated it would indicate that EGFR and/or AURKA inhibition are mandatory to support and maintain KRAS G12C inhibition. Partially this effect might be specific for the currently used G12C inhibitor (ARS1620) but due to similar binding mode as with AMG 520 it seems that this conclusion will be a general one (Janes et al., 2018). Besides of AMG 520 and MRTX849 there are also two other KRAS G12C targeting inhibitors, namely JNJ-74699157 (NCT04006301) and LY349946 (NCT04165031), in the clinical trials.

## Conclusions and Future Remarks

History has shown us that it is indeed not straight-forward to develop transcription or translation inhibitors. Even more difficult is to target these inhibitors only toward malignant cells. There have been a few clinical successes in their development, such as the CDK inhibitors, especially in combination with other chemotherapeutics. Targeting these central cellular processes has advantages to be more directed to cancer cells than non-specific chemotherapeutic agents such as cisplatin. On the side of disadvantages, targeting transcription and translation may affect multiple pathways hence circumventing the targeted pathway, and there are inevitable side effects arising from the fact that all cells require transcription and translation for their proper function. Transcription and translation are fundamental processes that targeting them is bound to result in cell death, as such cancer treatment based on these processes needs to be done in a manner that is safe for healthy cells. To achieve the specific and effective treatment, the myriad of proteins involved will continue to offer drug design possibilities far into the future. In addition, RNA was previously considered to be undruggable and not suitable as a drug itself, but now RNA-targeting and RNA-based drugs can be used as very precise methods to target some cancers. The recently discovered RNA activation of transcription offers uncharted possibilities in the treatment of cancer. Furthermore, transcription factors and oncogenes were also thought to be undruggable, but within the last few years we have seen some molecules targeting them entering clinical trials. By widening the scope of drug targets from traditional proteins with specified binding sites to transcription factors, oncogenes and RNA molecules, we are discovering new and specific ways to target cancer cells. So far, we have produced some highly specific, safe and efficacious cancer therapies which inhibit transcription and translation, and the newly discovered targets and our ever-increasing knowledge about the biological basics of these processes is bound to keep this field of inhibitor development ongoing far into the future.

## Author Contributions

NL-K, GP, AP, and PK wrote sections and participated in the creation of the graphics of the manuscript. All authors contributed to manuscript revision, read and approved the submitted version.

## Conflict of Interest

The authors declare that the research was conducted in the absence of any commercial or financial relationships that could be construed as a potential conflict of interest.

## Abbreviations

ASO: Antisense oligonucleotide
AURKA: Aurora kinase A
BERA: Bioengineered non-coding RNA agent
BET: Bromodomain and extraterminal domain
CDK: Cyclin-dependent kinase
EGFR: Epidermal growth factor receptor
Pol: RNA polymerase
RAS: Rat sarcoma viral oncogene homolog
RISC: RNA-induced silencing complex
RNAi: RNA interference.
